# Overcoming Challenges in Small-Molecule Drug Bioavailability: A Review of Key Factors and Approaches

**DOI:** 10.3390/ijms252313121

**Published:** 2024-12-06

**Authors:** Ke Wu, Soon Hwan Kwon, Xuhan Zhou, Claire Fuller, Xianyi Wang, Jaydutt Vadgama, Yong Wu

**Affiliations:** 1 Division of Cancer Research and Training, Department of Internal Medicine, Charles R. Drew University of Medicine and Science, Los Angeles, CA 90095, USA; 2 David Geffen School of Medicine at University of California, Los Angeles, CA 90095, USA; 3 Department of Pre-Biology, University of California, Santa Barbara (UCSB), Santa Barbara, CA 93106, USA; 4 Department of Whiting School of Engineering, Johns Hopkins University, Baltimore, MD 21218, USA; 5 Department of Chemistry, University of Illinois Urbana-Champaign, Urbana, IL 61801, USA

**Keywords:** bioavailability, ADME, artificial intelligence, pharmaceutical technology, predictive modeling, structural modification

## Abstract

The bioavailability of small-molecule drugs remains a critical challenge in pharmaceutical development, significantly impacting therapeutic efficacy and commercial viability. This review synthesizes recent advances in understanding and overcoming bioavailability limitations, focusing on key physicochemical and biological factors influencing drug absorption and distribution. We examine cutting-edge strategies for enhancing bioavailability, including innovative formulation approaches, rational structural modifications, and the application of artificial intelligence in drug design. The integration of nanotechnology, 3D printing, and stimuli-responsive delivery systems are highlighted as promising avenues for improving drug delivery. We discuss the importance of a holistic, multidisciplinary approach to bioavailability optimization, emphasizing early-stage consideration of ADME properties and the need for patient-centric design. This review also explores emerging technologies such as CRISPR-Cas9-mediated personalization and microbiome modulation for tailored bioavailability enhancement. Finally, we outline future research directions, including advanced predictive modeling, overcoming biological barriers, and addressing the challenges of emerging therapeutic modalities. By elucidating the complex interplay of factors affecting bioavailability, this review aims to guide future efforts in developing more effective and accessible small-molecule therapeutics.

## 1. Introduction

Small-molecule drugs remain the cornerstone of modern pharmacotherapy, representing over 90% of FDA-approved therapeutics [1]. However, poor oral bioavailability continues to be a major hurdle in small-molecule drug development, contributing significantly to high attrition rates in clinical trials [2,3]. Bioavailability—defined as the fraction of an administered dose that reaches systemic circulation—is a critical determinant of a drug’s therapeutic efficacy, safety profile, and commercial viability [2,4].

The multifaceted challenges associated with achieving adequate oral bioavailability stem from the complex interplay of physicochemical properties, formulation characteristics, and physiological barriers that govern a drug’s journey from ingestion to its site of action [5,6]. Key factors influencing bioavailability include aqueous solubility, gastrointestinal permeability, first-pass metabolism, and efflux transport mechanisms [7,8]. The “Rule of Five” proposed by Lipinski et al. provided initial guidelines for designing orally bioavailable drugs [9], but the pharmaceutical landscape has evolved to recognize the limitations of such simplified rules and the need for more nuanced, mechanism-based approaches [10,11].

Recent advances in medicinal chemistry, drug delivery technologies, and computational modeling have expanded the toolbox available to overcome bioavailability challenges [12,13]. Strategies range from molecular design modifications and prodrug approaches to innovative formulation techniques like nanocarriers and amorphous solid dispersions [14,15]. Additionally, the emergence of artificial intelligence and machine learning has accelerated the prediction and optimization of drug-like properties, including bioavailability [16,17].

This review provides a comprehensive examination of the key factors influencing small-molecule bioavailability and the cutting-edge approaches being employed to enhance it. We begin by dissecting the physicochemical and biological determinants of bioavailability, including solubility, lipophilicity, molecular size, intestinal permeability, metabolic stability, and the role of efflux transporters. We then explore state-of-the-art strategies for bioavailability enhancement, encompassing both the structural modifications of drug molecules and advanced formulation techniques.

The review also delves into the rapidly evolving field of computational tools and artificial intelligence in bioavailability prediction and optimization. We discuss in silico models for ADME (absorption, distribution, metabolism, and excretion) properties, physiologically based pharmacokinetic (PBPK) modeling, and the integration of machine learning approaches with traditional predictive methods. Case studies highlighting the successful applications of these strategies in drug discovery and development are presented to illustrate real-world impact.

Finally, we examine emerging technologies and future directions in the field, including personalized medicine approaches to bioavailability optimization, the potential of the gut microbiome in modulating drug absorption, and the challenges and opportunities presented by novel modalities like oligonucleotides and peptides.

By synthesizing the current state of knowledge and identifying key trends and challenges, this review aims to provide a roadmap for researchers and pharmaceutical scientists working to overcome bioavailability hurdles in small-molecule drug development. As the complexity of drug targets increases and the boundaries of druggable space expand, innovative approaches to enhancing bioavailability will play a crucial role in unlocking the full potential of small-molecule therapeutics and addressing unmet medical needs.

## 2. Key Factors Influencing Small-Molecule Bioavailability

Recent advances in pharmaceutical sciences have revealed the complex interplay of multiple factors governing small-molecule bioavailability. This section critically examines the key determinants of bioavailability, analyzing how physicochemical properties, biological barriers, and molecular interactions collectively influence drug absorption and distribution. Understanding these fundamental factors has become increasingly crucial for rational drug design and development strategies (Table 1).

### 2.1. Physicochemical Properties

**Figure 1:**
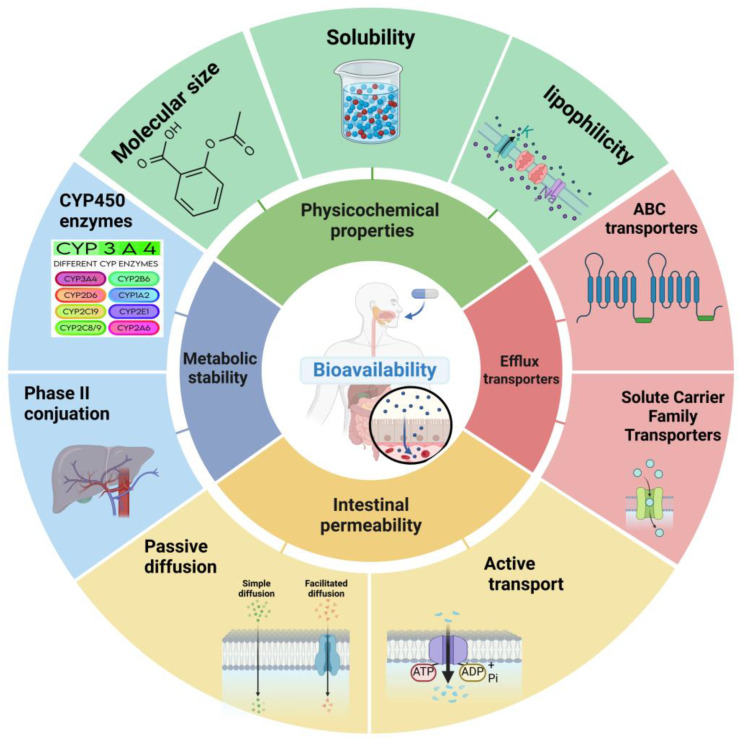
Key factors influencing small-molecule drug bioavailability. The bioavailability of small-molecule drugs is influenced by a complex interplay of factors. The central circle represents bioavailability, surrounded by four key determinants: physicochemical properties, intestinal permeability, metabolic stability, and efflux transporters. Physicochemical properties, including solubility and lipophilicity, affect a drug’s ability to dissolve and permeate biological membranes. Intestinal permeability determines the extent of drug absorption through the gastrointestinal tract. Metabolic stability influences the fraction of the drug that survives first-pass metabolism, while efflux transporters can actively pump drugs out of cells, potentially limiting their absorption and distribution. Understanding and optimizing these factors is crucial for enhancing the bioavailability of small-molecule drugs.

The physicochemical properties of small molecules play a pivotal role in determining their bioavailability. These inherent characteristics influence how a drug interacts with biological systems, affecting its solubility, permeability, and overall pharmacokinetic profile [18,19]. Understanding and optimizing these properties is crucial for developing orally bioavailable drugs (Figure 1).

#### 2.1.1. Solubility

Solubility represents a critical physicochemical property that fundamentally determines drug absorption and bioavailability [20,21,22,23]. For effective absorption through the gastrointestinal (GI) tract, drugs must first achieve adequate dissolution in the aqueous environment of the GI lumen, with poor aqueous solubility often resulting in incomplete absorption and reduced bioavailability. The Biopharmaceutics Classification System (BCS), introduced by Amidon et al., provides a framework for categorizing drugs based on their solubility and permeability characteristics, helping predict rate-limiting steps in drug absorption and guide formulation strategies [24,25]. According to BCS criteria, high solubility is defined when the highest dose strength dissolves in 250 mL or less of aqueous media across the physiological pH range of 1.0–7.5 at 37 °C.

Recent advances in computational chemistry have enabled a more accurate prediction of aqueous solubility. Quantitative structure–property relationship (QSPR) models, molecular dynamics simulations, and machine learning approaches are increasingly being used to estimate solubility during early-stage drug design [26,27]. These in silico methods allow for the rapid screening of large compound libraries and guide the selection of candidates with optimal solubility profiles.

Strategies to enhance solubility include the following:

(a) Salt formation: For ionizable compounds, creating salt forms can significantly improve aqueous solubility. The selection of appropriate counterions is critical, as it affects not only solubility but also stability and hygroscopicity [28].
(b) Cocrystals: Pharmaceutical cocrystals, composed of a drug molecule and one or more non-toxic coformers, can enhance solubility by altering crystal packing and intermolecular interactions [29].
(c) Amorphous solid dispersions: Dispersing the drug in an amorphous state within a polymer matrix can increase the apparent solubility and dissolution rate [15,30].
(d) Particle size reduction: Nanonization techniques, such as wet-milling or high-pressure homogenization, can dramatically increase the specific surface area of drug particles, enhancing dissolution rates [31].

#### 2.1.2. Lipophilicity

Lipophilicity, often quantified by the logarithm partition coefficient (logP) or logarithm distribution coefficient (logD), is a measure of a compound’s ability to dissolve in fats, oils, and non-polar solvents [32,33]. It plays a crucial role in determining a drug’s ability to permeate biological membranes, including the intestinal epithelium [34].

The relationship between lipophilicity and oral bioavailability is not linear. An optimal range of lipophilicity exists for achieving maximum oral bioavailability [35]. This concept is reflected in Lipinski’s Rule of Five, which suggests that compounds with a logP ≤ 5 are more likely to have good oral bioavailability [9].

Recent studies have refined our understanding of the optimal lipophilicity range:

- A logP between 1 and 3 is generally considered favorable for oral bioavailability, balancing membrane permeability with aqueous solubility [36].
- For central nervous system (CNS) drugs, a slightly higher logP range (2–4) may be optimal due to the need to cross the blood–brain barrier [37].
- The concept of ligand-lipophilicity efficiency (LLE) has emerged as a useful metric in drug design, combining potency and lipophilicity to guide optimization efforts [38].

Advances in computational methods have improved our ability to predict and optimize lipophilicity. Fragment-based approaches and machine learning models trained on large datasets of experimental logP values have enhanced the accuracy of in silico lipophilicity predictions [39,40].

#### 2.1.3. Molecular Size and Weight

Molecular size is another critical factor influencing a drug’s ability to passively diffuse through biological membranes [41,42]. As molecular size increases, the rate of passive diffusion tends to decrease, potentially limiting oral bioavailability [43].

Lipinski’s Rule of Five suggests that compounds with a molecular weight ≤ 500 Da are more likely to have good oral bioavailability [9]. However, this is not an absolute cutoff, and many successful drugs exceed this limit, particularly those that utilize active transport mechanisms or have high potency [44].

Recent analyses have provided more nuanced insights into the relationship between molecular size and bioavailability:

- An even lower molecular-weight cutoff of around 300–350 Da might be optimal for achieving high oral bioavailability, especially when considering factors like metabolic stability and clearance [9].
- The concept of molecular complexity, which considers not only size but also the presence of rigid structures and chiral centers, has emerged as a more comprehensive predictor of oral bioavailability [45].
- For macrocycles and other large molecules that violate traditional drug-likeness rules, specific structural features such as intramolecular hydrogen bonding and conformational flexibility can enable unexpectedly high oral bioavailability [46].

Advanced computational tools, including molecular dynamics simulations and machine learning models, are increasingly being used to predict the impact of molecular size and shape on membrane permeability and oral bioavailability [47,48]. These methods allow for more accurate predictions and can guide the design of molecules that balance size with other crucial physicochemical properties.

In conclusion, the physicochemical properties of solubility, lipophilicity, and molecular size/weight are interconnected factors that significantly influence small-molecule bioavailability. Understanding and optimizing these properties is crucial in drug discovery and development to enhance the likelihood of achieving adequate bioavailability and, ultimately, therapeutic efficacy. As our understanding of these properties continues to evolve, and new computational tools emerge, we can expect further refinements in our ability to design highly bioavailable small-molecule drugs.

### 2.2. Biological Factors

While physicochemical properties establish the foundation for a drug’s bioavailability potential, biological factors play an equally critical role in determining the ultimate fate of an orally administered small molecule. These biological factors involve complex interactions between the drug and various physiological processes and structures within the body, significantly influencing ADME profiles. Understanding these factors is crucial for designing drugs with optimal bioavailability profiles and for developing strategies to overcome biological barriers to absorption.

#### 2.2.1. Intestinal Permeability

Intestinal permeability stands as a critical determinant of oral drug bioavailability, representing the ability of a drug to traverse the intestinal epithelium and enter the bloodstream [49,50]. The mechanisms underlying intestinal drug absorption are diverse and can significantly impact a drug’s bioavailability profile.

Passive transcellular diffusion remains the primary route for many small-molecule drugs, especially lipophilic compounds. In this process, molecules diffuse through the lipid bilayer of epithelial cells, with the rate of diffusion largely dependent on the drug’s lipophilicity and molecular size [34,51]. This mechanism underscores the importance of optimizing physicochemical properties during drug design to enhance passive permeability.

For small, hydrophilic molecules, paracellular diffusion offers an alternative route of absorption. This pathway involves the passage of molecules through the tight junctions between epithelial cells. However, this route is generally limited to compounds with molecular weights below 200–250 Da and is particularly important for peptides and other hydrophilic molecules [52]. The restricted nature of this pathway highlights the challenges faced in developing orally bioavailable drugs for larger, hydrophilic molecules.

Active transport mechanisms play a crucial role in the absorption of certain drugs, often enabling the uptake of compounds that would otherwise have poor permeability. Key transporters include peptide transporters (e.g., PEPT1) for peptide-like drugs and prodrugs [53], organic anion-transporting polypeptides (OATPs) for various organic anions [54], and glucose transporter 1 (GLUT1) for glucose-conjugated drugs [55]. The exploitation of these transport systems has emerged as a promising strategy for enhancing the bioavailability of drugs that are poor candidates for passive diffusion.

Recent advances in understanding intestinal drug absorption have shed light on additional factors influencing permeability. The role of the unstirred water layer as a barrier to absorption, particularly for lipophilic compounds, has gained recognition [56]. This layer, which lies adjacent to the intestinal epithelium, can significantly impact the dissolution and subsequent absorption of poorly water-soluble drugs. Additionally, the impact of intestinal mucus on drug diffusion and absorption has been elucidated, revealing its dual role as both a barrier and a facilitator of drug transport [57].

**Figure 2:**
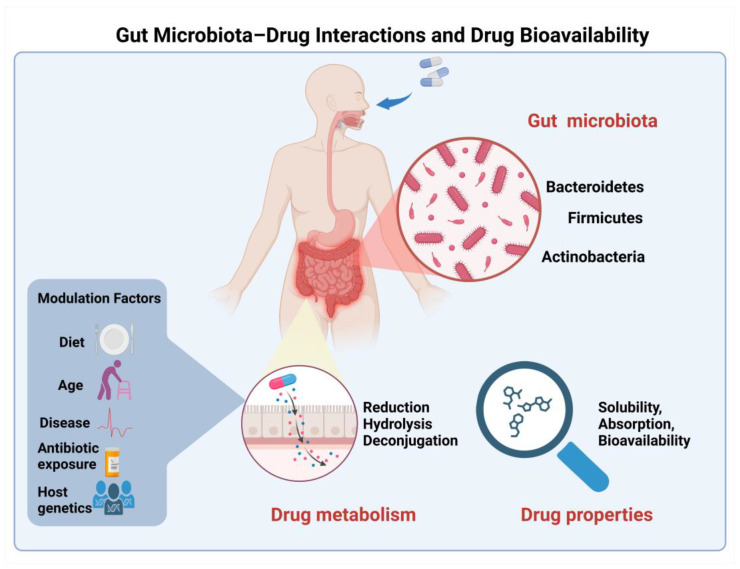
Schematic representation of gut microbiota–drug interactions in the intestinal lumen and their impact on drug bioavailability. Major bacterial phyla (Firmicutes, Bacteroidetes, and Actinobacteria) contribute to drug metabolism through direct mechanisms including reduction, hydrolysis, and deconjugation reactions. Multiple factors, including diet, age, disease state, antibiotic exposure, and host genetics, modulate these microbiota–drug interactions. The collective impact of these interactions ultimately influences key drug properties including solubility, absorption, and overall bioavailability.

The influence of the gut microbiome on drug metabolism and absorption has emerged as a fascinating area of research [58]. The complex interplay between gut bacteria and drug molecules can lead to altered bioavailability through various mechanisms, including the direct metabolism of drugs by bacterial enzymes and the modulation of host metabolic pathways (Figure 2). This growing field of research promises to unveil new strategies for enhancing drug bioavailability through microbiome modulation.

The gut microbiome has emerged as a pivotal determinant in drug bioavailability, fundamentally influencing drug delivery optimization. Recent advances in microbiome research have elucidated multiple mechanisms through which gut microbiota modulate drug absorption and metabolism [59]. Direct drug metabolism occurs through several key pathways in the gut’s anaerobic environment. Reductive metabolism, exemplified by the bacterial transformation of sulfasalazine to sulfapyridine and 5-aminosalicylic acid, represents a crucial biotransformation pathway essential for therapeutic efficacy [60]. Hydrolytic reactions, particularly those mediated by bacterial β-glucuronidases, play a vital role in drug reactivation and enterohepatic recycling, as demonstrated in irinotecan metabolism [61]. Additionally, deconjugation reactions facilitated by microbial enzymes significantly affect compound bioavailability, notably in the metabolism of dietary polyphenols through deglycosylation processes.

Beyond direct metabolic effects, gut microbiota exerts indirect influences on drug absorption through various mechanisms. Bacterial metabolites can modulate local intestinal pH, consequently affecting drug solubility and absorption patterns [62]. Microbial products demonstrate significant influence on epithelial barrier function through the modification of tight junction proteins, thereby impacting drug permeability [58]. Furthermore, bacterial products can compete with drugs for host metabolic enzymes, introducing an additional layer of complexity to drug bioavailability regulation [62].

The influence of gut microbiota on drug bioavailability exhibits marked inter-individual variations, governed by multiple factors. These variations stem from dietary patterns, age-related changes in microbiome composition, underlying disease states, historical antibiotic exposure, and genetic factors that shape the host–microbiome interaction. This complex interplay underscores the importance of considering microbiome status in drug development and therapeutic optimization strategies.

Accurate assessment of intestinal permeability is crucial for predicting oral bioavailability, and several models have been developed to this end. The Caco-2 cell monolayer model remains the gold standard for in vitro permeability assessment. These human colon adenocarcinoma cells form tight junctions and express many of the transporters found in the human small intestine [63]. Recent refinements to this model include the development of co-culture systems incorporating mucus-secreting cells for more physiologically relevant assessments [64] and the use of induced pluripotent stem cell (iPSC)-derived intestinal epithelial cells to capture genetic diversity [65].

Complementing cell-based assays, the parallel artificial membrane permeability assay (PAMPA) offers a non-cell-based approach to assess passive permeability. Recent improvements in this technique include the development of biomimetic lipid mixtures that better mimic the intestinal membrane composition [66], enhancing the physiological relevance of the model.

Advanced 3D culture systems, such as organoids and organ-on-a-chip models, have emerged as promising alternatives for permeability assessment. These systems provide a more physiologically relevant environment that includes multiple cell types and fluid flow [67,68], potentially offering more predictive power than traditional 2D cultures.

In situ perfusion models, involving the perfusion of a segment of the intestine in anesthetized animals, continue to provide valuable insights into drug absorption. Recent developments in this field include the use of imaging techniques to visualize drug transport in real-time [69,70], offering unprecedented insights into the dynamics of intestinal absorption.

While in vivo pharmacokinetic studies provide the most comprehensive assessment of intestinal absorption and overall bioavailability, their resource-intensive nature limits their use in high-throughput screening [71]. Nevertheless, these studies remain crucial for validating findings from in vitro and ex vivo models and for understanding the complex interplay of factors influencing bioavailability in living systems.

#### 2.2.2. Metabolic Stability

Metabolic stability, defined as a drug’s resistance to biotransformation by metabolic enzymes, plays a pivotal role in determining bioavailability. This factor is particularly critical for orally administered drugs that undergo first-pass metabolism in the intestine and liver before reaching systemic circulation [72].

The cytochrome P450 (CYP) enzymes stand as the vanguard of drug metabolism, responsible for the oxidative biotransformation of a vast array of small molecules. Recent research has expanded our understanding of CYP-mediated metabolism beyond the liver, highlighting the role of extra-hepatic CYP enzymes in drug disposition [73]. Moreover, the impact of genetic polymorphisms in CYP enzymes on drug metabolism and bioavailability has gained significant attention, offering insights into interindividual variability in drug response and toxicity [74].

UDP-glucuronosyltransferases (UGTs) represent another crucial class of drug-metabolizing enzymes, catalyzing the conjugation of glucuronic acid to a wide range of substrates. Recent advances in this field include the development of selective UGT inhibitors as probes for studying drug metabolism [75], providing valuable tools for dissecting the complex pathways of drug biotransformation. Additionally, the emerging role of the gut microbiome in modulating UGT activity [76] has opened new avenues for understanding and potentially manipulating drug metabolism to enhance bioavailability.

Phase II enzymes, including sulfotransferases, N-acetyltransferases, and glutathione S-transferases, have gained recognition for their importance in the metabolism of drugs that are not CYP substrates [77]. Recent research has also highlighted the potential for drug–drug interactions mediated by these enzymes [78], underscoring the need for a comprehensive understanding of metabolic pathways when predicting and optimizing drug bioavailability.

To enhance metabolic stability and improve bioavailability, several innovative strategies have been developed. Structural modifications to block or alter metabolically labile sites on drug molecules remain a cornerstone approach. Recent advances in this area include the application of computational methods to predict metabolic hot spots and guide rational drug design [79]. The use of bioisosteres to replace metabolically labile groups while maintaining drug-like properties has also proven effective in many cases [80].

Deuteration, the replacement of hydrogen atoms with deuterium, has emerged as a powerful tool for enhancing metabolic stability. The approval of deutetrabenazine as the first deuterated drug demonstrates the clinical potential of this approach [81]. Ongoing research is exploring the synergistic effects of combining deuteration with other metabolic stability enhancement strategies [82], potentially offering more robust solutions to metabolic instability.

Prodrug approaches continue to evolve as sophisticated strategies for overcoming metabolic barriers to bioavailability. Recent innovations in this field include the development of site-specific enzyme-activated prodrugs for targeted drug delivery [83], allowing for precise control over the location and timing of drug activation. Additionally, the integration of prodrug strategies with nanocarrier-based delivery systems has shown promise in overcoming multiple barriers to bioavailability simultaneously [84].

The complex interplay between metabolic stability and other factors influencing bioavailability underscores the need for integrated approaches to drug design and development. As our understanding of metabolic processes deepens and new tools for predicting and modulating drug metabolism emerge, we can anticipate more targeted and effective strategies for enhancing the bioavailability of metabolically labile compounds.

#### 2.2.3. Efflux Transporters

Efflux transporters represent a significant biological barrier to drug absorption and distribution, playing a crucial role in the bioavailability of many small molecules. These membrane-bound proteins actively pump drugs out of cells, potentially limiting their absorption and altering their pharmacokinetic profiles. While P-glycoprotein (P-gp) remains the most well-characterized efflux transporter, others such as the breast cancer resistance protein (BCRP) and multidrug resistance-associated proteins (MRPs) also significantly impact drug disposition [85,86,87].

The influence of efflux transporters on drug bioavailability is multifaceted. In the intestine, these proteins can substantially reduce net drug absorption by pumping molecules back into the lumen, effectively creating a barrier to oral bioavailability [88]. This process is particularly impactful for drugs that are substrates for multiple efflux transporters or those with low passive permeability. Moreover, the expression of efflux transporters in the liver and kidneys can enhance drug clearance, further reducing systemic exposure [89,90]. The blood–brain barrier presents an additional challenge, with its high expression of efflux transporters limiting the central nervous system penetration of many therapeutics.

Recent research has expanded our understanding of efflux transporters beyond these established roles. Studies have revealed their importance in tissue-specific drug disposition and toxicity, highlighting how transporter expression patterns can lead to unexpected drug accumulation or exclusion in certain organs [91]. The impact of genetic polymorphisms in efflux transporter genes on drug response and bioavailability has also gained attention, offering insights into interindividual variability in drug efficacy and toxicity [92]. Furthermore, the complex interplay between drug metabolism and efflux transport has emerged as a critical determinant of overall bioavailability, with some metabolites serving as transporter substrates or inhibitors, thereby altering the disposition of the parent compound [93].

To overcome the challenges posed by efflux transporters, researchers have developed several innovative strategies. The structural modifications of drug molecules represent a direct approach to mitigating transporter-mediated efflux. Recent advances in this area include the application of machine learning models to predict the substrate specificity of efflux transporters, guiding rational drug design to reduce transporter affinity [94]. The development of dual inhibitors that target both efflux transporters and metabolic enzymes has also shown promise, offering a multifaceted approach to enhancing bioavailability [95].

Prodrug strategies have gained traction as an elegant solution to efflux transporter-mediated limitations on bioavailability. By designing prodrugs that are poor substrates for efflux transporters but that can be converted to the active form after absorption, researchers can effectively bypass this biological barrier [96]. This approach has been particularly successful for drugs targeting the central nervous system, where efflux transporters at the blood–brain barrier pose a significant challenge.

The co-administration of transporter inhibitors remains a viable strategy for enhancing drug absorption and distribution. Recent developments in this field have focused on identifying safer alternatives to synthetic inhibitors, with natural products emerging as promising candidates [97]. These compounds often exhibit multi-target effects, potentially offering broader spectrum inhibition of drug efflux mechanisms. Additionally, the development of nanocarrier-based systems for co-delivery of drugs and efflux transporter inhibitors has shown potential in preclinical studies, allowing for targeted inhibition at the site of drug absorption [98].

Formulation strategies represent another frontier in overcoming efflux transporter-mediated limitations on bioavailability. Lipid-based nanocarriers have demonstrated significant potential in enhancing the oral bioavailability of efflux transporter substrates [99]. These systems can not only protect drugs from efflux but also enhance their solubility and permeability. Moreover, the development of stimuli-responsive nanoparticles offers a sophisticated approach to avoiding efflux transporter interactions. These advanced delivery systems can release their payload in response to specific physiological cues, such as pH changes or enzymatic activity, potentially circumventing efflux mechanisms altogether [100].

The ongoing research into efflux transporters and strategies to mitigate their impact on drug bioavailability underscores the complexity of this biological barrier. As our understanding of transporter biology deepens and technologies for drug design and delivery advance, we can anticipate more targeted and effective approaches to enhancing the bioavailability of efflux transporter substrates. The integration of computational methods, advanced formulation techniques, and mechanistic insights into transporter function promises to yield novel solutions to this longstanding challenge in drug development.

In conclusion, biological factors including intestinal permeability, metabolic stability, and efflux transport significantly influence the bioavailability of small-molecule drugs. The intricate interplay between these factors necessitates a multifaceted approach to bioavailability enhancement, combining insights from molecular biology, pharmacology, and pharmaceutical technology. As our knowledge of these biological processes continues to expand, and new technologies emerge, we can expect further innovations in overcoming biological barriers to drug absorption and distribution. These advancements will not only improve the efficacy of existing therapeutics but also open new avenues for developing drugs that were previously limited by poor bioavailability.

## 3. Strategies to Enhance Small-Molecule Bioavailability

**Figure 3:**
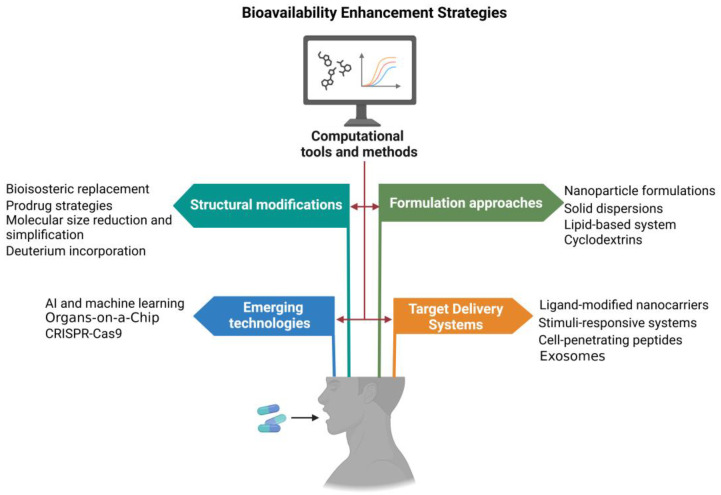
Advanced strategies and technologies for enhancing drug bioavailability. Structural modifications, such as bioisosteric replacement, molecular size reduction, etc., are employed to optimize the physicochemical properties of drugs. Formulation approaches, including nanoparticle formulations, lipid-based systems, etc., focus on enhancing drug solubility and stability. Targeted delivery systems, such as ligand-modified nanocarriers, exosomes, etc., are designed for precise delivery to specific tissues, reducing off-target effects. Emerging technologies, including AI, machine learning, organ-on-a-chip models, etc., provide innovative platforms for developing and refining these bioavailability enhancing strategies.

The optimization of small-molecule bioavailability remains a critical challenge in drug development, often determining the success or failure of promising therapeutic candidates. Recent years have witnessed a surge in innovative approaches to overcome the multifaceted barriers to oral bioavailability, leveraging advances in medicinal chemistry, pharmaceutical technology, and computational modeling. These strategies span a broad spectrum, from rational structural modifications of drug molecules to cutting-edge formulation techniques and targeted delivery systems (Figure 3). This section explores the most promising and impactful methodologies for enhancing small-molecule bioavailability, examining their underlying principles, recent breakthroughs, and potential for clinical translation (Table 2). By integrating insights from molecular design, advanced materials science, and physiological understanding, these approaches are reshaping the landscape of drug delivery and expanding the horizons of oral therapeutics.

### 3.1. Formulation Approaches

Formulation strategies play an instrumental role in optimizing the bioavailability of small-molecule therapeutics by systematically addressing physicochemical and biological barriers to drug absorption and distribution. These approaches have proven particularly valuable for compounds with challenging solubility and permeability profiles, enabling significant advances in drug delivery optimization. As our understanding of drug delivery systems advances, formulation scientists continue to develop innovative techniques that can significantly improve the pharmacokinetic profiles of challenging drug candidates.

One of the most fundamental and widely employed formulation strategies is the enhancement of drug solubility. For poorly water-soluble drugs, which constitute a significant proportion of new chemical entities, improving solubility is often the key to unlocking their therapeutic potential. Salt formation remains a cornerstone technique in this regard, particularly effective for ionizable compounds. The selection of an appropriate counterion is critical, as it can affect not only solubility but also stability, hygroscopicity, and other important properties [101]. Recent advances in this field include the development of computational tools for predicting salt formation propensity and stability, allowing for a more rational selection of counterions.

Building upon the principles of salt formation, the development of cocrystals has emerged as a sophisticated approach to solubility enhancement. Pharmaceutical cocrystals, composed of a drug molecule and one or more non-toxic coformers, can improve solubility by altering the crystal packing and intermolecular interactions of the drug [30]. The flexibility in coformer selection allows for the fine-tuning of physicochemical properties beyond what is possible with traditional salt forms. Recent research has focused on developing predictive models for cocrystal formation and stability, as well as exploring the use of natural products as coformers to improve the overall safety profile of formulations.

Amorphous solid dispersions (ASDs) represent another powerful tool in the formulator’s toolkit. By dispersing the drug in an amorphous state within a polymer matrix, ASDs can dramatically increase apparent solubility and dissolution rates [15]. The success of products like Gris-PEG^®^ (griseofulvin dispersed in polyethylene glycol) and Sporanox^®^ (itraconazole dispersed in hydroxypropyl methylcellulose) has spurred further research in this area. Recent innovations include the development of novel polymer carriers with enhanced stability and drug-loading capacity, as well as the application of hot-melt extrusion and spray-drying technologies for more efficient ASD production.

Lipid-based formulations, such as self-emulsifying drug delivery systems (SEDDS) and lipid nanoparticles, have gained significant traction for enhancing the bioavailability of lipophilic drugs. These formulations typically consist of the drug dissolved in a mixture of oils, surfactants, and co-solvents, which form fine oil-in-water emulsions upon contact with gastrointestinal fluids [102]. The success of products like Neoral^®^ (cyclosporine in a SEDDS formulation) has demonstrated the clinical potential of this approach. Recent research has focused on developing “super-SEDDS” with enhanced drug-loading capacity and stability, as well as exploring the use of naturally derived lipids to improve biocompatibility.

Recent advances in polymer technology have significantly expanded the toolbox for enhancing drug bioavailability. Novel block copolymers incorporating functional groups such as PEG-PLGA with terminal carboxyl groups have shown improved drug–polymer interactions and the enhanced stability of amorphous solid dispersions [103]. Smart copolymers responding to specific physiological triggers, such as pH-sensitive poly(2-diisopropylaminoethyl methacrylate) derivatives, have demonstrated precise control over drug release in specific gastrointestinal regions [104,105].

Nanotechnology has opened new avenues for bioavailability enhancement, with nanocrystal formulations standing at the forefront. By reducing drug particles to nanoscale dimensions, typically through techniques like wet-milling or high-pressure homogenization, nanocrystal formulations can dramatically increase the specific surface area of drug particles, enhancing the dissolution rates [106]. The success of products like Tricor^®^ (nanocrystalline fenofibrate) has spurred further research in this area. Recent innovations include the development of combination nanocrystal formulations, incorporating multiple poorly soluble drugs in single-dosage form, and the exploration of surface-modified nanocrystals for targeted drug delivery.

The field of nanocrystal technology has also seen significant advancement. Sophisticated milling processes, including combination approaches of wet- and cryo-milling, have enabled the production of nanocrystals with enhanced stability and narrow size distributions. Surface-modified nanocrystals, particularly those functionalized with targeting ligands or stabilizing polymers, have shown improved tissue-specific delivery and reduced aggregation tendency [107]. For instance, recent work utilizing surface modification with hyaluronic acid has demonstrated enhanced mucoadhesive properties and improved oral bioavailability of poorly soluble drugs [108].

Cyclodextrins have long been recognized for their ability to form inclusion complexes with poorly soluble drugs, effectively increasing their apparent solubility. Recent research has expanded on this concept, developing modified cyclodextrins with enhanced complexation efficiency and reduced toxicity. The success of products like Sporanox^®^ (itraconazole complexed with hydroxypropyl-β-cyclodextrin) has demonstrated the clinical viability of this approach. Current research is exploring the use of cyclodextrin-based nanocarriers and stimuli-responsive cyclodextrin complexes for controlled drug release.

The advent of 3D-printing technologies has opened up new possibilities in personalized medicine and complex formulations. Additionally, 3D-printed dosage forms offer the potential for customized dosing and release profiles, which could optimize bioavailability for individual patients [109,110]. Recent advances include the development of multi-material 3D-printing techniques for creating dosage forms with complex internal structures and composition gradients, allowing for precise control over drug release kinetics.

Absorption enhancers represent another important class of formulation excipients aimed at improving bioavailability. These compounds can work through various mechanisms, including increasing membrane fluidity, opening tight junctions between epithelial cells, or inhibiting efflux transporters [111]. Recent research has focused on developing safer and more effective absorption enhancers, including naturally derived compounds and synthetic peptides designed to transiently modulate epithelial barrier function.

As formulation approaches continue to evolve, there is an increasing emphasis on combination strategies that address multiple barriers to bioavailability simultaneously. For example, lipid-based nanocarriers incorporating absorption enhancers and enzyme inhibitors can potentially improve solubility, permeability, and metabolic stability in a single formulation. The development of such multifunctional delivery systems represents a promising frontier in bioavailability enhancement.

In conclusion, formulation approaches offer a diverse and powerful set of tools for enhancing the bioavailability of small-molecule drugs. As our understanding of drug delivery systems deepens and new technologies emerge, we can anticipate even more sophisticated and effective formulation strategies. The continued integration of computational modeling, advanced manufacturing techniques, and mechanistic insights into drug absorption and distribution promises to yield novel solutions to longstanding bioavailability challenges, ultimately improving therapeutic outcomes for patients.

### 3.2. Structural Modifications

Structural modifications represent a powerful approach to improving the bioavailability of small-molecule drugs. This strategy aims to optimize the physicochemical properties of the drug molecule itself, addressing issues such as poor solubility, low permeability, or metabolic instability. The art of structural modification lies in enhancing bioavailability while maintaining or improving the compound’s pharmacological activity and safety profile.

Bioisosteric replacement is a cornerstone technique in medicinal chemistry for optimizing drug-like properties, including bioavailability. This approach involves replacing a functional group of a molecule with another group that has similar physical or chemical properties. The principle of bioisosterism is based on the observation that certain atomic or molecular groups with similar size, shape, and electronic properties can often be interchanged while maintaining or improving the biological activity of the compound [112]. In the context of bioavailability enhancement, bioisosteric replacements can be employed to improve solubility, enhance metabolic stability, modify lipophilicity, or alter pKa.

Classical bioisosteres, such as the replacement of hydrogen with fluorine or a hydroxyl group with an amino group, have long been used to fine-tune drug properties. However, the field has evolved to embrace more complex, non-classical bioisosteres that can dramatically alter a compound’s pharmacokinetic profile. For instance, the replacement of a carboxylic acid group with a tetrazole moiety has been successfully employed in the development of several angiotensin II receptor antagonists, including losartan [113]. This modification not only improved oral bioavailability but also enhanced metabolic stability.

Recent advances in computational chemistry have greatly enhanced our ability to predict and design effective bioisosteric replacements. Machine learning algorithms trained on large datasets of known bioisosteres can now suggest novel replacements that might not be immediately obvious to medicinal chemists [114]. These in silico approaches are complemented by high-throughput screening methods that can rapidly assess the impact of various bioisosteric replacements on key pharmacokinetic parameters.

Prodrug strategies represent another powerful approach to enhancing bioavailability through structural modification. A prodrug is an inactive precursor of a drug that is converted to the active form in vivo, typically through enzymatic or chemical processes. This approach can address various bioavailability issues by temporarily masking polar groups, adding ionizable moieties, or creating more lipophilic derivatives [115].

Ester prodrugs are among the most common types, owing to their relative ease of synthesis and the ubiquity of esterases in the body. A classic example is oseltamivir (Tamiflu^®^), an ethyl ester prodrug of oseltamivir carboxylate, which significantly improves oral bioavailability [116]. The success of oseltamivir has inspired the development of numerous ester prodrugs, with ongoing research focusing on site-specific esterases for targeted drug activation.

Phosphate prodrugs have gained prominence, particularly for improving the aqueous solubility of poorly soluble compounds intended for parenteral administration. Fosphenytoin, a phosphate ester prodrug of phenytoin, exemplifies this approach, offering improved solubility for intravenous use [117]. Recent research in phosphate prodrugs has expanded to include more complex phosphoramidate derivatives, which can enhance cellular uptake and improve oral bioavailability of nucleoside analogs and other challenging drug classes.

The concept of molecular size reduction has emerged as a valuable strategy for enhancing bioavailability, particularly in the context of oral absorption. This approach is rooted in the observation that smaller molecules generally have better permeability across biological membranes. Fragment-based drug discovery (FBDD) has gained traction as a method for developing drug candidates with optimized physicochemical properties, including enhanced bioavailability [118].

FBDD starts with very small molecules (fragments) and builds them up to create drug candidates. This approach often results in compounds with lower molecular weight and better physicochemical properties compared to traditional high-throughput screening hits. The success of vemurafenib, a BRAF inhibitor developed using FBDD, demonstrates the potential of this approach in creating orally bioavailable drugs for challenging targets [119].

Molecular truncation and simplification represent complementary strategies to FBDD. These approaches involve systematically removing non-essential parts of a molecule to reduce its size and complexity while maintaining or improving its biological activity. The development of trimethoprim from pyrimethamine illustrates the power of this approach, resulting in a smaller molecule with improved solubility and bioavailability [120].

Recent advances in structural modification strategies have been greatly facilitated by the integration of artificial intelligence and machine learning techniques. These computational tools can analyze vast databases of structure–activity and structure–property relationships to suggest modifications likely to improve bioavailability while maintaining target affinity. Generative models, in particular, have shown promise in designing novel molecular structures with optimized properties [12].

The application of deuterium incorporation as a means of enhancing metabolic stability and, consequently, bioavailability has gained significant attention. By replacing key hydrogen atoms with deuterium, the rate of certain metabolic reactions can be slowed, potentially leading to improved pharmacokinetic profiles. The approval of deutetrabenazine for the treatment of chorea associated with Huntington’s disease marked a milestone in this field, demonstrating the clinical viability of deuteration strategies [121].

As our understanding of drug–target interactions and pharmacokinetics deepens, more sophisticated structural modification strategies are emerging. The design of peptidomimetics and macrocycles that can achieve oral bioavailability despite violating traditional drug-like property rules represents an exciting frontier in medicinal chemistry [122]. These approaches often involve the careful manipulation of intramolecular hydrogen bonding and conformational flexibility to balance membrane permeability with target affinity.

In conclusion, structural modifications offer a diverse and powerful set of tools for enhancing the bioavailability of small-molecule drugs. The success of this approach lies in the judicious application of medicinal chemistry principles, guided by advanced computational tools and a deep understanding of structure–property relationships. As the field continues to evolve, we can anticipate even more sophisticated and effective strategies for optimizing drug bioavailability through structural design. The integration of these approaches with advanced formulation techniques and targeted delivery systems promises to unlock the full therapeutic potential of small-molecule drugs, addressing unmet medical needs and improving patient outcomes.

## 4. Computational Tools and Methods for Bioavailability Prediction

### 4.1. In Silico Models for ADME Properties

The development of in silico models for predicting ADME properties has revolutionized the early stages of drug discovery and development. These computational approaches offer a rapid and cost-effective means of estimating how drug candidates will interact with biological systems, significantly reducing the time and resources required for experimental testing. As the pharmaceutical industry continues to face challenges in bringing new drugs to market, the role of in silico ADME modeling has become increasingly critical in streamlining the drug discovery process and improving the success rate of candidates progressing through clinical trials.

Quantitative structure–property relationship (QSPR) models form the cornerstone of many in silico ADME prediction tools. These mathematical models relate the structural features of molecules to their physicochemical or biological properties, providing a powerful framework for predicting key parameters that influence bioavailability. In the context of ADME prediction, QSPR models are commonly employed to estimate properties such as solubility, lipophilicity (logP/logD), intestinal permeability, and plasma protein binding [123].

The development of QSPR models typically involves several key steps. Initially, a diverse set of molecular descriptors is calculated, encompassing topological, electronic, and geometrical properties of the compounds under study. These descriptors serve as the foundation for capturing the structural nuances that influence ADME properties. Subsequently, sophisticated statistical methods, including multiple linear regression, partial least squares, and machine learning algorithms, are employed to establish relationships between these descriptors and the ADME properties of interest. The final step involves rigorous validation of the model using external datasets to ensure its predictive power and applicability domain [124].

Recent advancements in QSPR modeling have seen the integration of more complex molecular representations and machine learning techniques. Graph neural networks, for instance, have shown promise in capturing the intricate structural details of molecules, leading to the improved predictions of ADME properties [125]. Moreover, the advent of deep learning architectures has enabled the automatic extraction of relevant features from molecular structures, potentially uncovering novel structure–property relationships that may not be apparent through traditional descriptor-based approaches [126]

One of the most significant applications of QSPR models in bioavailability prediction has been in estimating human intestinal absorption. Models have been developed that can predict the fraction of an orally administered dose that reaches the systemic circulation, taking into account factors such as solubility, permeability, and first-pass metabolism [127]. These models have proven invaluable in early-stage drug discovery, allowing researchers to prioritize compounds with favorable absorption profiles for further development.

Another crucial application of QSPR models is in predicting Caco-2 cell permeability, which serves as a surrogate for intestinal permeability. These models have become increasingly sophisticated, incorporating not only passive diffusion parameters but also considering the influence of active transport mechanisms and efflux pumps [128]. By providing rapid estimates of intestinal permeability, these models enable researchers to optimize molecular structures for enhanced oral bioavailability early in the drug design process.

While QSPR models offer valuable insights into individual ADME properties, physiologically based pharmacokinetic (PBPK) modeling represents a more holistic approach to predicting drug disposition in the body. PBPK models integrate physicochemical properties of drugs with detailed physiological parameters to simulate drug concentrations in various tissues over time. These models typically consist of multiple compartments representing different organs and tissues, connected by blood flow, providing a mechanistic framework for understanding and predicting complex pharmacokinetic behaviors [129].

The power of PBPK modeling lies in its ability to incorporate a wide range of physiological and drug-specific parameters. These may include organ volumes, blood flow rates, tissue partition coefficients, and clearance rates. By integrating this diverse set of inputs, PBPK models can generate detailed concentration–time profiles for drugs in different tissues, offering insights into bioavailability, drug–drug interactions, and potential toxicity risks [130].

One of the key advantages of PBPK modeling in bioavailability prediction is its ability to account for the complex interplay between different ADME processes. For instance, these models can simulate the impact of first-pass metabolism on oral bioavailability, taking into account both intestinal and hepatic metabolism. This level of detail allows researchers to identify rate-limiting steps in drug absorption and distribution, guiding targeted optimization efforts [131].

PBPK models have also proven particularly valuable in predicting bioavailability under different physiological conditions. By adjusting model parameters to reflect variations in gastric emptying time, intestinal transit time, or pH, researchers can simulate drug absorption in fed versus fasted states or in different patient populations. This capability is especially useful in designing clinical trials and optimizing dosing regimens for diverse patient groups [132].

The integration of in vitro data into PBPK models has further enhanced their predictive power. Techniques such as in vitro–in vivo extrapolation (IVIVE) allow researchers to incorporate data from cell-based assays or microsomal preparations into PBPK models, bridging the gap between laboratory experiments and whole-body pharmacokinetics. This approach has been particularly successful in predicting drug–drug interactions mediated by cytochrome P450 enzymes, a critical factor in assessing the bioavailability and safety of combination therapies [133].

As computational power has increased and modeling techniques have advanced, the pharmaceutical industry has seen a rise in the use of commercial PBPK modeling software packages. Platforms such as Simcyp, developed by Certara and based in Princeton, USA, GastroPlus from Simulations Plus in Lancaster, USA, and PK-Sim, part of the OSP Suite and developed in Basel, Switzerland, have gained widespread adoption. These platforms offer user-friendly interfaces and extensive databases of physiological parameters, which help streamline model development and applications in drug discovery and development. More information about these tools can be found on their official websites: www.certara.com, www.simulations-plus.com, and www.open-systems-pharmacology.org. These tools have democratized access to sophisticated PBPK modeling capabilities, enabling the broader application of these techniques across drug discovery and development pipelines [134].

Looking to the future, the integration of artificial intelligence and machine learning approaches with traditional QSPR and PBPK modeling techniques promises to further enhance our ability to predict and optimize drug bioavailability. These advanced computational methods offer the potential to uncover complex, non-linear relationships in ADME data and to generate novel hypotheses for improving drug absorption and distribution. As these tools continue to evolve, they will undoubtedly play an increasingly central role in guiding the design and development of the next generation of small-molecule therapeutics.

### 4.2. Machine Learning and Artificial Intelligence Approaches

The rapid advancement of machine learning (ML) and artificial intelligence (AI) technologies has ushered in a new era in bioavailability prediction and optimization. These sophisticated computational approaches offer unprecedented capabilities in analyzing complex datasets, identifying subtle patterns, and generating predictive models that can significantly accelerate the drug discovery and development process.

Deep learning, a subset of machine learning based on artificial neural networks, has emerged as a particularly powerful tool in predicting various ADME properties, including bioavailability. The key advantage of deep learning models lies in their ability to automatically learn complex features from raw data, potentially uncovering relationships that might be overlooked by traditional statistical methods. Convolutional neural networks (CNNs), for instance, have shown remarkable success in predicting human oral bioavailability based on molecular graphs [135]. These models can process 2D or 3D representations of molecules directly, eliminating the need for hand-crafted descriptors and potentially capturing more nuanced structural information relevant to bioavailability.

Another promising application of deep learning in bioavailability prediction is the use of long short-term memory (LSTM) networks for modeling sequential ADME data [136]. These recurrent neural network architectures are particularly well suited for analyzing time-series data, such as drug concentration profiles over time. By training on large datasets of pharmacokinetic studies, LSTM models can learn to predict complex absorption and distribution patterns, offering insights into how structural modifications might impact bioavailability over time.

The integration of multiple data sources and models represents a frontier in AI-driven bioavailability prediction. Modern AI approaches often leverage diverse datasets to improve predictive accuracy and robustness. These data integrations can include experimental results from different assays (e.g., solubility, permeability, metabolic stability), literature-derived information, and high-throughput screening data. By combining these heterogeneous data sources, AI models can develop a more comprehensive understanding of the factors influencing bioavailability, leading to more accurate and generalizable predictions [137].

Ensemble methods, which combine predictions from multiple models, have shown particular promise in enhancing the reliability of bioavailability predictions. Techniques such as random forests, gradient boosting, and stacking can aggregate insights from various predictive models, including traditional QSAR models, physics-based simulations, and neural networks. These ensemble approaches often outperform individual models by capturing different aspects of the complex relationships governing bioavailability [138].

Transfer learning has emerged as a powerful paradigm in AI-driven bioavailability prediction, especially when dealing with limited data for specific drug classes or ADME properties. This approach allows models trained on large, diverse datasets to be fine-tuned for more specific prediction tasks, even when data for those tasks are scarce. For example, a model initially trained to predict general drug absorption properties could be adapted to predict the bioavailability of a particular class of compounds with minimal additional data [139]. This capability is particularly valuable in early-stage drug discovery, where experimental data may be limited.

The application of generative models represents an exciting frontier in AI-driven drug design for enhanced bioavailability. Techniques such as variational autoencoders (VAEs) and generative adversarial networks (GANs) can be trained on large databases of known drugs and their bioavailability profiles to generate novel molecular structures with optimized properties. These models can explore vast chemical spaces efficiently, proposing new compounds that balance target affinity with favorable ADME characteristics, including high bioavailability [140].

Explainable AI (XAI) techniques are gaining importance in the field of bioavailability prediction, addressing the “black box” nature of many complex ML models. Methods such as SHAP (SHapley Additive exPlanations) values and LIME (Local Interpretable Model-agnostic Explanations) allow researchers to understand which molecular features or properties are most influential in a model’s predictions. This interpretability is crucial not only for building trust in AI-driven predictions but also for guiding medicinal chemists in rational drug design for improved bioavailability [141].

The integration of AI with high-performance computing and automated experimentation platforms is paving the way for closed-loop optimization of drug bioavailability. In these systems, AI models can propose structural modifications or formulation strategies to enhance bioavailability, which are then rapidly tested through automated synthesis and screening platforms. The results of these experiments feed back into the AI models, continuously refining their predictions and accelerating the optimization process [142].

Despite the remarkable progress in AI-driven bioavailability prediction, several challenges remain. Data quality and quantity continue to be critical factors limiting the performance of ML models. Efforts to standardize ADME data reporting and to create large, diverse, and well-curated datasets are ongoing and will be crucial for further advancements in the field. Additionally, ensuring the generalizability of AI models across diverse chemical spaces and their ability to make reliable predictions for novel molecular scaffolds remains an active area of research [143].

As we look to the future, the integration of AI with other emerging technologies, such as quantum computing and blockchain, may offer new avenues for enhancing bioavailability prediction and optimization. Quantum machine learning algorithms, for instance, could potentially handle the high-dimensional spaces of molecular descriptors more efficiently, leading to more accurate predictions. Meanwhile, blockchain technology could facilitate the secure sharing of proprietary ADME data across the pharmaceutical industry, dramatically expanding the datasets available for training AI models [144].

**Figure 4:**
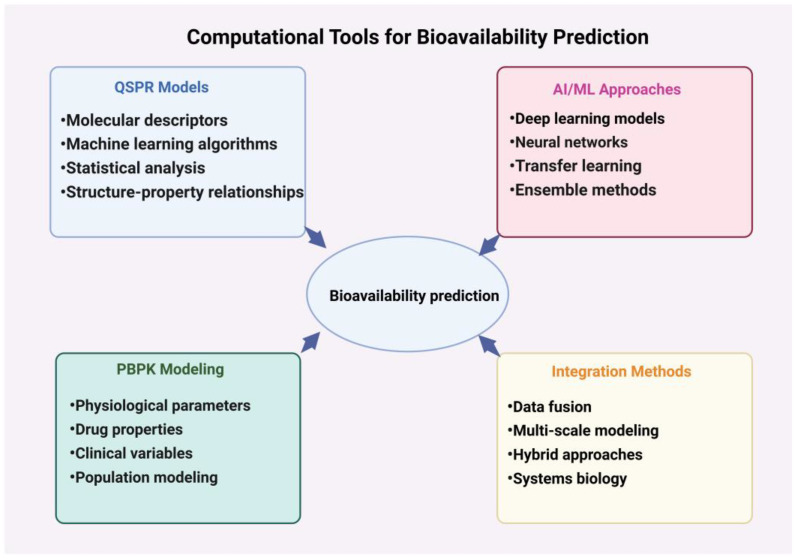
Schematic representation of computational tools and methods used in bioavailability prediction. This figure illustrates four main approaches: QSPR models (structure–property relationships), AI/ML approaches (artificial intelligence and machine learning), PBPK modeling (physiologically based pharmacokinetic modeling), and integration methods. Arrows indicate the interconnected nature of these approaches and their collective contribution to bioavailability prediction.

To sum up, machine learning and artificial intelligence approaches have transformed the landscape of bioavailability prediction, offering unprecedented capabilities in analyzing complex pharmacokinetic data and guiding drug design (Figure 4). As these technologies continue to evolve and integrate with other cutting-edge tools, they promise to accelerate the discovery and development of small-molecule drugs with optimized bioavailability profiles, ultimately leading to more effective and safer therapeutics for patients.

## 5. Case Studies and Success Stories

### 5.1. Examples of Small Molecules with Improved Bioavailability Through Formulation Optimization

The field of pharmaceutical formulation has witnessed remarkable successes in enhancing the bioavailability of small-molecule drugs. These case studies not only demonstrate the power of innovative formulation strategies but also provide valuable insights into overcoming specific bioavailability challenges. By examining these success stories, we can glean principles that may guide future efforts in drug delivery optimization.

Itraconazole, a broad-spectrum antifungal agent, represents a classic example of how formulation can dramatically improve bioavailability. The drug’s extremely low aqueous solubility (less than 1 μg/mL at neutral pH) initially posed a significant barrier to its oral effectiveness. To address this challenge, researchers developed a solid dispersion formulation using hydroxypropyl methylcellulose (HPMC) as a carrier. This innovative approach led to the creation of Sporanox^®^, an oral solution that demonstrated a remarkable 27% increase in bioavailability compared to the conventional capsule formulation [145]. The success of Sporanox^®^ illustrates the potential of solid dispersion techniques in enhancing the dissolution and absorption of poorly soluble drugs.

The story of Sporanox^®^ also highlights the importance of understanding the physicochemical properties of both the drug and the carrier polymer. The interaction between itraconazole and HPMC not only improved solubility but also helped maintain the drug in a supersaturated state in the gastrointestinal tract, further enhancing absorption. This case underscores the need for a mechanistic understanding of drug–polymer interactions in the design of effective solid dispersion formulations.

Another compelling example of formulation-driven bioavailability enhancement is seen in the development of Tricor^®^ (fenofibrate). Fenofibrate, a lipid-lowering agent, initially suffered from poor and variable bioavailability due to its low aqueous solubility. The breakthrough came with the application of nanocrystal technology, achieved through wet-milling techniques. By reducing the particle size to the nanoscale, the specific surface area of fenofibrate was dramatically increased, leading to enhanced dissolution rates and improved absorption [146].

The nanocrystalline formulation of fenofibrate not only improved bioavailability but also allowed for lower doses and administration without regard to food intake. This case demonstrates the potential of nanotechnology in addressing bioavailability challenges and improving patient convenience. Moreover, the success of Tricor^®^ spurred further research into nanocrystal formulations, leading to the development of several other commercially successful products.

The journey of ritonavir, an HIV protease inhibitor, provides insights into the challenges and solutions in maintaining consistent bioavailability. The initial formulation of ritonavir (Norvir^®^) faced stability issues, with the formation of a less-soluble crystalline form during storage leading to reduced bioavailability. To overcome this challenge, Abbott Laboratories developed a melt-extruded solid dispersion formulation [147].

This new formulation not only improved stability but also maintained consistent bioavailability and reduced the pill burden for patients. The case of ritonavir highlights the importance of considering long-term stability in formulation design, particularly for drugs prone to polymorphic transformations. It also demonstrates the potential of hot-melt extrusion technology in creating stable amorphous solid dispersions.

The development of a self-emulsifying drug delivery system (SEDDS) for cyclosporine, resulting in the product Neoral^®^, represents another landmark in bioavailability enhancement through formulation. Cyclosporine, an immunosuppressant with poor aqueous solubility, showed significant improvements in bioavailability and reduced pharmacokinetic variability when formulated as a SEDDS [148]. The success of Neoral^®^ not only improved therapeutic outcomes for transplant patients but also paved the way for the wider adoption of lipid-based formulations in addressing bioavailability challenges.

The case of aprepitant (Emend^®^), an antiemetic drug, illustrates the synergy between structural modification and formulation optimization. While structural changes, including the addition of a trifluoromethyl group, improved the drug’s inherent properties, the use of nanoparticle technology in the final formulation further enhanced its bioavailability. This combination approach resulted in a product with improved solubility, reduced first-pass metabolism, and enhanced oral bioavailability [149].

These case studies collectively highlight several key principles in formulation-driven bioavailability enhancement:

1. The importance of a thorough understanding of the drug’s physicochemical properties in guiding formulation strategy.
2. The power of nanotechnology in addressing solubility and dissolution-rate limited absorption.
3. The potential of amorphous solid dispersions in maintaining drugs in a high-energy state for improved dissolution.
4. The value of lipid-based formulations for enhancing the absorption of lipophilic drugs.
5. The need for considering long-term stability in formulation design.
6. The benefits of combining multiple strategies, including structural modification and advanced formulation techniques.

As the field of pharmaceutical formulation continues to evolve, these success stories serve as inspiration and guidance for addressing the bioavailability challenges of future drug candidates. They underscore the critical role of innovative formulation approaches in unlocking the full therapeutic potential of small-molecule drugs, ultimately leading to improved patient outcomes and more effective medications.

### 5.2. Case Studies of Structural Modifications Leading to Enhanced Bioavailability

The strategic modification of molecular structures has proven to be a powerful approach in enhancing the bioavailability of small-molecule drugs. These case studies not only showcase the ingenuity of medicinal chemists but also provide valuable insights into the principles governing drug absorption and distribution. By examining these success stories, we can derive key lessons that may guide future efforts in drug design and optimization.

Oseltamivir, marketed as Tamiflu^®^, stands as a prime example of how prodrug strategies can dramatically improve oral bioavailability. The active compound, oseltamivir carboxylate, exhibited poor oral bioavailability due to its polar nature, limiting its effectiveness as an antiviral agent. To overcome this challenge, researchers developed an ethyl ester prodrug (oseltamivir) that significantly enhanced oral absorption. The prodrug approach resulted in a remarkable increase in bioavailability, with oseltamivir demonstrating over 80% oral bioavailability compared to the negligible absorption of the parent compound [150].

The success of oseltamivir illustrates the power of prodrug design in masking polar groups to improve passive membrane permeability. By temporarily modifying the carboxylic acid moiety as an ethyl ester, the lipophilicity of the molecule was increased, facilitating its absorption through the intestinal epithelium. Once absorbed, the prodrug is rapidly hydrolyzed by esterases in the liver, releasing the active oseltamivir carboxylate. This case underscores the importance of considering both the absorption phase and the subsequent metabolic activation in prodrug design.

Another compelling example of structural modification enhancing bioavailability is seen in the development of tenofovir alafenamide (TAF), a next-generation prodrug of the antiviral agent tenofovir. The original prodrug, tenofovir disoproxil fumarate (TDF), while effective, faced challenges related to suboptimal bioavailability and potential renal toxicity. TAF was designed as a novel phosphonamidate prodrug to address these limitations [151].

The innovative design of TAF resulted in 4–7 times higher intracellular concentrations of the active metabolite at a fraction of the dose of TDF, with reduced systemic exposure and an improved renal safety profile. This case highlights the potential of advanced prodrug strategies in not only enhancing bioavailability but also improving the overall pharmacokinetic and safety profile of a drug. The success of TAF demonstrates how structural modifications can be leveraged to achieve targeted drug delivery, concentrating the active compound in desired tissues while minimizing systemic exposure.

These structural changes resulted in a compound with improved solubility, reduced first-pass metabolism, and enhanced oral bioavailability. The case of aprepitant illustrates the importance of considering multiple pharmacokinetic parameters simultaneously in drug design. By addressing both solubility and metabolic stability through strategic structural modifications, researchers were able to create a drug with superior bioavailability and efficacy.

The development of sitagliptin, a dipeptidyl peptidase-4 (DPP-4) inhibitor for the treatment of type 2 diabetes, offers another instructive example of structure-guided bioavailability enhancement. Initial lead compounds in this class suffered from poor oral bioavailability due to their high polar surface area and low lipophilicity. Through systematic structural modifications, including the incorporation of a fluorinated triazolopiperazine moiety, researchers at Merck were able to optimize the balance between potency and pharmacokinetic properties [152].

The resulting compound, sitagliptin, exhibited excellent oral bioavailability along with high potency and selectivity for DPP-4. This case underscores the importance of considering bioavailability early in the drug discovery process and demonstrates how structural modifications can be used to fine-tune multiple drug-like properties simultaneously.

The journey of dasabuvir, a non-nucleoside inhibitor of the hepatitis C virus NS5B polymerase, provides insights into the use of conformational restriction to enhance bioavailability. Early compounds in this series showed promising activity but poor pharmacokinetic properties. By introducing conformational constraints through the incorporation of a cyclopropyl ring, researchers were able to reduce the compound’s flexibility and improve its metabolic stability [153].

This structural modification not only enhanced the metabolic stability of dasabuvir but also improved its binding affinity to the target enzyme. The resulting compound demonstrated superior oral bioavailability and efficacy in clinical trials, leading to its approval as part of a combination therapy for hepatitis C. This case highlights the potential of conformational restriction as a strategy for optimizing both pharmacodynamic and pharmacokinetic properties.

These case studies collectively illuminate several key principles in structural modification for bioavailability enhancement:

1. The power of prodrug approaches in overcoming absorption barriers while maintaining the ability to deliver the active compound.
2. The importance of considering multiple pharmacokinetic parameters simultaneously in drug design.
3. The potential of strategic functional group modifications in fine-tuning lipophilicity, solubility, and metabolic stability.
4. The value of conformational restriction in optimizing both target-binding and pharmacokinetic properties.
5. The benefits of early consideration of bioavailability in the drug discovery process.

As medicinal chemists continue to push the boundaries of drug design, these success stories serve as valuable guides for addressing the bioavailability challenges of future drug candidates. They underscore the critical role of rational, structure-guided approaches in creating small-molecule drugs with optimal pharmacokinetic profiles, ultimately leading to more effective and safer therapies for patients.

### 5.3. Lessons Learned and Best Practices

The wealth of experience accumulated through numerous bioavailability enhancement efforts has yielded invaluable insights and best practices for drug discovery and development. These lessons, derived from both successes and failures, provide a roadmap for addressing bioavailability challenges in future pharmaceutical endeavors.

A key lesson that emerges from these case studies is the importance of an integrated approach to bioavailability enhancement. Successful strategies often combine multiple techniques, including both formulation optimization and structural modifications. The case of aprepitant (Emend^®^) exemplifies this approach, where structural changes to improve solubility and reduce first-pass metabolism were complemented by advanced formulation techniques [154]. This synergistic approach allowed for a more comprehensive solution to bioavailability challenges, addressing multiple limiting factors simultaneously.

The integrated approach extends beyond the laboratory, emphasizing the need for collaboration between medicinal chemists, formulation scientists, and pharmacologists. By fostering interdisciplinary teamwork, pharmaceutical companies can leverage diverse expertise to develop more effective strategies for enhancing bioavailability. This collaborative model enables a holistic view of drug development, considering not only the intrinsic properties of the drug molecule but also its interaction with delivery systems and biological environments.

Another critical lesson is the importance of addressing bioavailability issues early in the drug discovery and development process. Early consideration of bioavailability can significantly improve the chances of success and reduce the overall development costs. The development of sitagliptin illustrates this principle, where researchers at Merck incorporated bioavailability considerations into their lead optimization process from the outset [155]. By doing so, they were able to create a compound with both high target affinity and favorable pharmacokinetic properties, streamlining the development process and minimizing the risk of late-stage failures.

This proactive approach to bioavailability optimization involves integrating in silico predictions, high-throughput screening for ADME properties, and early pharmacokinetic studies into the drug discovery pipeline. By front-loading these assessments, researchers can identify and address potential bioavailability issues before significant resources are invested in a candidate’s development.

These case studies also highlight the importance of tailoring solutions to the specific challenges posed by each drug candidate. While general principles of bioavailability enhancement can guide efforts, the most successful approaches are often those that are carefully customized to the unique physicochemical properties and therapeutic goals of the compound in question. The development of tenofovir alafenamide (TAF) demonstrates this principle, where a novel prodrug strategy was designed to address the specific limitations of the original compound, resulting in improved tissue-specific delivery and reduced systemic exposure [156].

This tailored approach requires a deep understanding of the drug’s physicochemical properties, its target site of action, and the biological barriers it must overcome. It also involves careful consideration of the intended patient population and administration route, as these factors can significantly impact the choice of bioavailability enhancement strategy.

The power of leveraging advanced technologies in formulation and structural design has been a recurring theme in many success stories. From nanotechnology in the case of fenofibrate (Tricor^®^) to the use of solid dispersion techniques for itraconazole (Sporanox^®^), cutting-edge technologies have played a crucial role in overcoming bioavailability challenges [157,158]. As new technologies emerge, such as AI-driven drug design and 3D-printed dosage forms, their integration into bioavailability enhancement efforts will likely yield novel solutions to persistent challenges.

The importance of establishing robust in vitro–in vivo correlations (IVIVC) has been underscored by many case studies. The accurate prediction of in vivo performance based on in vitro data is crucial for streamlining the development process and reducing the need for extensive animal and human studies. The development of biorelevant dissolution methods and physiologically based pharmacokinetic (PBPK) modeling has greatly improved the ability to establish these correlations, as seen in the optimization of various solid dispersion formulations [159].

The consideration of manufacturing and scalability has emerged as a critical factor in the success of bioavailability enhancement strategies. Techniques that show promise in the laboratory must also be feasible for large-scale production and maintain long-term stability. The case of ritonavir (Norvir^®^) highlights this principle, where the development of a melt-extruded solid dispersion formulation not only improved bioavailability but also addressed manufacturing and stability challenges [160].

Finally, the importance of patient-centric design in bioavailability enhancement strategies cannot be overstated. Successful approaches not only improve the pharmacokinetic profile of a drug but also consider factors such as dosing frequency, administration route, and overall treatment burden. The development of extended-release formulations and fixed-dose combinations exemplifies this patient-focused approach, aiming to improve adherence and quality of life for patients.

In brief, these lessons and best practices provide a valuable framework for addressing bioavailability challenges in drug development. By embracing an integrated, proactive, and patient-centric approach, leveraging advanced technologies, and maintaining a focus on manufacturability and scalability, researchers can enhance their chances of developing successful, highly bioavailable drug products. As the field continues to evolve, these principles will undoubtedly be refined and expanded, guiding the next generation of pharmaceutical innovations.

## 6. Future Perspectives and Challenges

### 6.1. Emerging Technologies and Approaches for Bioavailability Enhancement

The landscape of bioavailability enhancement is rapidly evolving, driven by advances in materials science, nanotechnology, and computational methods. These emerging technologies and approaches promise to revolutionize how we address bioavailability challenges, offering new possibilities for improving drug efficacy and patient outcomes.

Stimuli-responsive nanocarriers represent a cutting-edge approach to enhancing bioavailability. These smart delivery systems are designed to release drugs in response to specific physiological conditions, such as pH, temperature, or enzymatic activity [161]. For instance, pH-sensitive nanocarriers can protect drugs from the harsh acidic environment of the stomach and selectively release their payload in the intestine, where absorption primarily occurs. The ability to fine-tune drug release based on physiological cues not only enhances bioavailability but also potentially reduces side effects by minimizing premature drug release in non-target tissues.

The application of 3D-printing technology in pharmaceutical manufacturing is opening new avenues for personalized medicine and complex drug delivery systems. Additionally, 3D-printed dosage forms offer unprecedented flexibility in tailoring drug release profiles and dosages to individual patient needs. Khan et al. have explored the potential of 3D printing in creating dosage forms with complex internal structures, allowing for precise control over drug release kinetics [162]. This technology enables the production of multi-layer tablets with varying drug concentrations or combinations of multiple drugs in a single dosage form, potentially improving bioavailability through optimized release patterns. Moreover, the ability to rapidly prototype and iterate formulations using 3D printing could significantly accelerate the drug development process.

Artificial intelligence (AI) and machine learning (ML) are increasingly being applied to drug design, including efforts to enhance bioavailability. These computational approaches can analyze vast datasets of molecular structures and their associated properties to identify patterns and relationships that may not be apparent through traditional methods. Qureshi et al. have demonstrated the potential of AI in predicting drug-like properties and guiding lead optimization, including the enhancement of bioavailability [163]. AI-driven platforms can suggest structural modifications to improve solubility, permeability, and metabolic stability while maintaining or enhancing therapeutic activity. This approach promises to streamline the drug discovery process and increase the success rate of candidates progressing through clinical trials.

The emergence of organ-on-a-chip and organoid technologies offers new possibilities for the more accurate prediction of drug absorption and bioavailability. These advanced in vitro models provide a more physiologically relevant environment for studying drug behavior compared to traditional cell culture systems. For instance, intestine-on-a-chip models can replicate the complex cellular architecture and fluid dynamics of the human gut, allowing for the more accurate assessment of drug permeability and first-pass metabolism. Bein et al. have shown how these models can be used to study the interplay between drug absorption, metabolism, and the gut microbiome, providing insights that could lead to novel strategies for enhancing bioavailability [164].

Genome-editing technologies, particularly CRISPR-Cas9, are opening up new avenues for modulating drug absorption and metabolism. While primarily explored in the context of disease treatment, these tools also have potential applications in enhancing drug bioavailability. Karlgren’s work has highlighted the possibility of using CRISPR-Cas9 to modulate the expression of drug-metabolizing enzymes or transporters, potentially tailoring bioavailability to individual genetic profiles [165]. This approach could lead to more personalized strategies for optimizing drug absorption and distribution, although ethical considerations and regulatory challenges will need to be carefully addressed.

The exploration of the gut microbiome’s role in drug metabolism and absorption is revealing new opportunities for enhancing bioavailability. Research by Zimmermann et al. has shown that the gut microbiota can significantly influence drug metabolism, either through the direct biotransformation of drug molecules or by modulating host metabolic pathways [166]. This growing understanding is leading to novel approaches for enhancing bioavailability, such as the development of probiotic formulations that can modulate the gut microbiome to favor drug absorption or the design of drugs that leverage microbial metabolism for activation or enhanced solubility.

Exosome-based drug delivery systems represent another promising frontier in bioavailability enhancement. These naturally occurring nanoparticles can be engineered to encapsulate drugs and target specific tissues, potentially improving both bioavailability and therapeutic efficacy. The work of Vader et al. has demonstrated the potential of exosomes in enhancing the delivery of small-molecule drugs and biologics across biological barriers [167]. Their ability to interact with cellular membranes and potentially bypass certain efflux mechanisms makes them an attractive option for improving the bioavailability of challenging drug candidates.

As these emerging technologies and approaches continue to develop, they promise to provide powerful new tools for addressing bioavailability challenges. However, their successful implementation will require overcoming various technical, regulatory, and ethical hurdles. Integrating these advanced technologies into existing drug development pipelines, ensuring their scalability and cost-effectiveness, and navigating the complex regulatory landscape for novel drug delivery systems will be critical challenges in the coming years. Nonetheless, the potential benefits in terms of improved drug efficacy, reduced side effects, and more personalized treatments make these emerging approaches highly promising avenues for future research and development in the field of bioavailability enhancement.

### 6.2. Addressing the Challenges of Poorly Soluble and Poorly Permeable Compounds

The persistent challenge of developing effective therapies from poorly soluble and poorly permeable compounds continues to drive innovation in the pharmaceutical industry. As our understanding of the physicochemical properties governing drug absorption deepens, novel approaches are emerging to tackle these longstanding issues.

Ionic liquids have garnered significant attention as a promising solution for solubilizing poorly water-soluble drugs. These designer solvents, composed of organic cations and anions, offer unique properties that can be tailored to enhance drug solubility and stability. Recent work by Marrucho et al. has demonstrated the potential of ionic liquids in creating highly concentrated, stable formulations of hydrophobic drugs [168]. The ability to fine-tune the properties of ionic liquids by altering their constituent ions provides a versatile platform for addressing solubility challenges across a wide range of drug molecules. Moreover, some ionic liquids have shown the ability to modulate drug permeability across biological membranes, potentially addressing both solubility and permeability issues simultaneously.

Mesoporous silica nanoparticles represent another cutting-edge approach to enhancing the solubility and dissolution rate of poorly water-soluble drugs. These materials offer exceptionally high surface areas and tunable pore sizes, thus allowing for efficient drug loading and controlled release. The work of Mäkilä et al. has shown how mesoporous silica can be used to create supersaturated drug solutions, significantly enhancing the apparent solubility and absorption of hydrophobic compounds [169]. The ability to functionalize the surface of these nanoparticles offers additional opportunities for improving drug stability and targeting specific absorption sites in the gastrointestinal tract.

For compounds with poor membrane permeability, cell-penetrating peptides have emerged as a powerful tool for enhancing intracellular delivery. These short peptide sequences can facilitate the transport of various cargo molecules, including drugs, across biological membranes. Bechara and Sagan have demonstrated the potential of cell-penetrating peptides in improving the oral bioavailability of peptide drugs and other poorly permeable compounds [170]. The diversity of naturally occurring and synthetic cell-penetrating peptides provides a rich palette for designing tailored delivery systems for specific drug molecules and target tissues.

The development of advanced permeation enhancers continues to push the boundaries of what is possible in oral drug delivery. Tight junction modulators in particular have shown promise in reversibly opening the paracellular pathway for enhanced absorption of hydrophilic drugs. The work of Maher et al. on fatty acid-based permeation enhancers has demonstrated significant improvements in the oral bioavailability of various therapeutic peptides [171]. However, the challenge remains to develop permeation enhancers that provide a suitable balance between efficacy and safety, ensuring enhanced drug absorption without compromising the integrity of the intestinal barrier.

Combination strategies that address both solubility and permeability issues simultaneously represent a frontier in bioavailability enhancement. Hybrid nanocarriers, such as lipid–polymer nanoparticles, offer the potential to improve drug solubility through encapsulation while also enhancing permeability through interactions with biological membranes. The work of Zhang et al. has shown how these hybrid systems can be designed to provide sustained drug release and improved oral bioavailability for poorly soluble and poorly permeable compounds [172].

Co-delivery systems that combine the drug with bioavailability enhancers or metabolism inhibitors in a single formulation are gaining traction as a sophisticated approach to improving oral absorption. For instance, the co-formulation of a poorly permeable drug with a P-glycoprotein inhibitor can address efflux-mediated limitations on absorption. Lomovskaya et al. have demonstrated the potential of this approach in enhancing the bioavailability of antibiotic compounds that are substrates for efflux transporters [173].

The application of nanotechnology to create “nanosized” drug particles continues to evolve, with new techniques emerging to produce stable nanocrystal formulations with enhanced dissolution properties. Advanced milling techniques and controlled crystallization methods are enabling the production of nanocrystals with narrow size distributions and tailored surface properties. The work of Müller et al. has shown how these refined nanocrystal formulations can significantly improve the oral bioavailability of drugs with poor aqueous solubility [174].

As these novel approaches continue to develop, the integration of computational methods for predicting and optimizing drug–excipient interactions is becoming increasingly important. Molecular dynamics simulations and machine learning algorithms are being employed to design more effective formulations for poorly soluble and poorly permeable compounds. The work of Gao et al. demonstrates how these computational tools can guide the selection of excipients and predict the performance of complex formulations, streamlining the development process for challenging drug candidates [175].

While these emerging technologies and approaches offer exciting possibilities for addressing the challenges of poorly soluble and poorly permeable compounds, several hurdles remain. Ensuring the long-term stability of advanced formulations, developing scalable manufacturing processes for complex delivery systems, and navigating the regulatory landscape for novel excipients and nanotechnology-based products are critical challenges that must be addressed.

Moreover, the translation of promising preclinical results to clinical success remains a significant hurdle. Improving the predictive power of in vitro and animal models for human drug absorption and developing more physiologically relevant screening tools will be crucial in bridging this gap.

As the field continues to evolve, a multidisciplinary approach combining expertise in pharmaceutics, materials science, and biophysics will be essential for developing the next generation of solutions for poorly soluble and poorly permeable compounds. By leveraging these emerging technologies and approaches, the pharmaceutical industry is poised to unlock the therapeutic potential of a wider range of drug candidates, ultimately leading to more effective treatments for patients.

### 6.3. Balancing Bioavailability with Other Drug-like Properties

The optimization of bioavailability, while crucial, must be carefully balanced with other essential drug-like properties to ensure the overall success of a therapeutic agent. This delicate balancing act represents one of the most significant challenges in modern drug discovery and development, requiring a holistic approach that considers multiple parameters simultaneously.

Integrating bioavailability considerations into early-stage drug design has become increasingly important as the pharmaceutical industry seeks to improve the efficiency of the drug discovery process. Multi-parameter optimization (MPO) strategies have emerged as powerful tools for addressing this challenge. These approaches aim to simultaneously optimize multiple properties, including potency, selectivity, metabolic stability, and bioavailability, from the earliest stages of lead identification and optimization. The work of Segall et al. has demonstrated how MPO can be effectively implemented using computational tools to guide medicinal chemistry efforts toward compounds with a balanced profile of drug-like properties [176]. By incorporating bioavailability predictions alongside other crucial parameters in scoring functions, researchers can make more informed decisions about which compounds to synthesize and advance through the pipeline.

The development of high-throughput screening methods for ADME properties has greatly facilitated the early assessment of bioavailability potential. Techniques such as parallel artificial membrane permeability assay (PAMPA) and Caco-2 cell permeability assays allow for the rapid evaluation of compound permeability, while solubility assays and metabolic stability screens provide early insights into other key determinants of bioavailability. The integration of these assays into the lead optimization process, as described by Di et al., enables researchers to quickly identify and address potential bioavailability issues before significant resources are invested in a particular chemical series [177].

However, the challenge lies not only in measuring these properties but in understanding their interplay and making informed trade-offs. For instance, increasing lipophilicity to improve membrane permeability may negatively impact aqueous solubility or increase the likelihood of off-target effects. Similarly, structural modifications to enhance metabolic stability might adversely affect target binding affinity. Advanced computational models, such as those developed by Wager et al., are increasingly being employed to predict these complex relationships and guide the design of molecules with an optimal balance of properties [178].

The interplay between bioavailability and toxicity presents another critical consideration in drug design. While high bioavailability is generally desirable for maximizing the therapeutic effect, it can also increase the risk of systemic toxicity. Strategies for addressing this challenge include the development of targeted delivery systems to enhance bioavailability at the site of action while minimizing systemic exposure. The work of Langer and colleagues on nanoparticle-based drug delivery systems exemplifies this approach, demonstrating how engineered nanocarriers can improve the therapeutic index of potent compounds by enhancing their tissue-specific bioavailability [179].

Prodrug approaches offer another avenue for balancing bioavailability with safety considerations. By designing prodrugs that are preferentially activated at the target site, researchers can enhance local bioavailability while minimizing systemic exposure and potential off-target effects. The success of capecitabine, an orally administered prodrug of 5-fluorouracil, in improving the efficacy and tolerability of cancer treatment illustrates the potential of this strategy [180].

The consideration of drug–drug interactions in bioavailability enhancement strategies is becoming increasingly important as polypharmacy becomes more common, particularly in aging populations. Physiologically based pharmacokinetic (PBPK) modeling has emerged as a powerful tool for predicting complex drug–drug interactions that may affect bioavailability. The work of Rowland et al. demonstrates how these models can be used to simulate the impact of concomitant medications on drug absorption and disposition, guiding dosing decisions and formulation strategies [134].

Moreover, the design of interaction-resistant drugs that maintain consistent bioavailability in the presence of common co-medications or dietary factors is an area of growing interest. This approach requires a deep understanding of the molecular mechanisms underlying drug–drug interactions, including the role of metabolizing enzymes and transporters. The development of rivaroxaban, an oral anticoagulant designed to have minimal interactions with food and other drugs, exemplifies the potential of this strategy in creating more robust and patient-friendly medications [181].

As our understanding of the factors influencing bioavailability continues to grow, new opportunities for fine-tuning drug properties are emerging. The exploration of the role of the gut microbiome in drug metabolism and absorption, for instance, opens up possibilities for designing drugs that leverage microbial metabolism for improved bioavailability or reduced toxicity. Similarly, advances in our understanding of chronobiology and circadian rhythms are leading to the development of chrono-modulated drug delivery systems that can optimize bioavailability based on the body’s natural rhythms.

In conclusion, balancing bioavailability with other drug-like properties remains a complex challenge that requires a multifaceted approach. The integration of advanced computational tools, high-throughput screening methods, and a mechanistic understanding of drug behavior in the body is crucial for making informed decisions in the drug design process. As we move toward more personalized approaches to medicine, the ability to fine-tune drug properties, including bioavailability, to individual patient needs will become increasingly important. This will require continued innovation in drug design, formulation technologies, and predictive modeling to create therapeutics that offer the optimal balance of efficacy, safety, and patient convenience.

## 7. Conclusions

The field of small-molecule drug bioavailability has undergone remarkable transformation through the convergence of multiple scientific disciplines and technological advances. This review has revealed several critical insights that significantly impact drug development and therapeutic outcomes. The integration of artificial intelligence with traditional drug design approaches has revolutionized our ability to predict and optimize bioavailability at the early stages of drug development. Furthermore, the emerging understanding of the gut microbiome’s role in drug disposition has opened new avenues for enhancing therapeutic efficacy through microbiome-informed drug design and delivery strategies.

The evolution from empirical to rational approaches in addressing bioavailability challenges represents a fundamental shift in pharmaceutical development. Advanced formulation strategies, particularly those involving stimuli-responsive materials and targeted delivery systems, have demonstrated remarkable potential in overcoming traditional bioavailability barriers. The success of these approaches, exemplified by recent developments in polymer technology and nanocrystal formulations, underscores the importance of mechanistic understanding in drug delivery optimization. The integration of multiple enhancement strategies has emerged as a particularly promising approach. Notably, combining microbiome-targeted techniques with advanced formulation technologies offers unprecedented opportunities for optimizing drug bioavailability. This synergistic approach not only addresses traditional physicochemical limitations but also leverages biological factors to enhance therapeutic outcomes.

Looking forward, several key research areas emerge as critical for advancing the field. The development of AI-driven platforms incorporating microbiome data and patient-specific factors promises to enable personalized bioavailability optimization. The integration of advanced materials science with biological understanding may create sophisticated delivery systems responding to both physicochemical and biological cues. Furthermore, the exploration of novel strategy combinations, particularly those targeting multiple absorption barriers simultaneously, represents a promising direction for future research. The investigation of microbiome–drug interactions at a mechanistic level will enable the rational design of microbiome-aware drug delivery systems.

For pharmaceutical scientists and drug developers, this review emphasizes the importance of the early consideration of bioavailability optimization through integrated approaches combining computational prediction, advanced formulation, and biological understanding. The implementation of systematic screening approaches must account for both traditional physicochemical properties and emerging biological factors, while adopting patient-centric design principles that consider individual variability in drug absorption and metabolism. The future of bioavailability enhancement lies in the seamless integration of multiple disciplines, from materials science to microbiology, supported by advanced computational tools and emerging technologies. Success in this endeavor requires continued collaboration across scientific domains and persistent innovation in both theoretical understanding and practical implementation.

This holistic approach to bioavailability optimization promises to accelerate the development of more effective therapeutics while potentially reducing the development costs and improving patient outcomes. As we move forward, the field must maintain focus on translating these advanced concepts into practical solutions that can be effectively implemented in clinical settings. The convergence of multiple scientific disciplines, coupled with technological innovations, positions the field for significant advances in addressing the persistent challenge of drug bioavailability. 

## Figures and Tables

**Table 1:** Comprehensive classification and impact analysis of drug bioavailability determinants.

Factor Category	Specific Factors	Impact on Bioavailability
Physicochemical Properties	Solubility	Determines dissolution rate and maximum absorbable dose
	Lipophilicity (logP/logD)	Affects membrane permeability and distribution
	Molecular size/weight	Influences passive diffusion and active transport
	pKa	Affects ionization state and absorption
	Crystal form	Impacts dissolution rate and solubility
Biological Factors	Intestinal permeability	Controls rate and extent of absorption
	Metabolic stability	Determines first-pass metabolism and bioavailability
	Efflux transporters	Affects cellular uptake and retention
	Gut microbiota	Modulates drug metabolism and absorption
Physiological Factors	GI pH	Influences drug ionization and dissolution
	GI transit time	Affects absorption window and extent
	Blood flow	Impacts drug distribution and absorption
	Disease state	Modifies absorption and metabolism
Formulation Factors	Particle size	Affects dissolution rate
	Excipients	Influences solubility and stability
	Dosage form	Determines release pattern and site
	Manufacturing process	Impacts physical properties and stability

**Table 2:** Detailed solutions and mechanisms for common bioavailability challenges.

Challenge	Enhancement Strategy	Mechanism	Example Applications
Poor Solubility	Salt formation	Formation of ionic species	Itraconazole mesylate
	Cocrystallization	Modified crystal packing	Carbamazepine-saccharin
	Amorphous solid dispersions	Enhanced dissolution rate	Gris-PEG^®^
	Nanocrystal formation	Increased surface area	Tricor^®^
Low Permeability	Lipid-based formulations	Enhanced lymphatic transport	Neoral^®^
	Permeation enhancers	Modulation of tight junctions	Sodium caprate
	Prodrug approaches	Improved membrane transport	Oseltamivir
	Nanocarrier systems	Enhanced cellular uptake	Abraxane^®^
Metabolic Instability	Deuteration	Reduced metabolic clearance	Deutetrabenazine
	Enzyme inhibitors	Reduced first-pass effect	Ritonavir combinations
	Site-specific delivery	Bypass first-pass metabolism	Transdermal systems
	Structural modification	Blocked metabolic sites	Sitagliptin
Efflux Transport	P-gp inhibitors	Reduced efflux	Verapamil
	Novel delivery systems	Efflux bypass	SEDDS
	Structural optimization	Reduced transporter affinity	Modified analogs
